# Mapping the “Ghost Fleet of Mallows Bay”, Maryland with drone-based remote sensing

**DOI:** 10.1038/s41597-025-05635-z

**Published:** 2025-09-25

**Authors:** Elizabeth C. White, Alexander C. Seymour, Julian Dale, Everette Newton, David W. Johnston

**Affiliations:** 1 Division of Marine Science and Conservation, Nicholas School of the Environment, Duke University Marine Laboratory, 135 Duke Marine Lab Rd, Beaufort, NC 28516 USA; 2 Formerly Division of Marine Science and Conservation, Nicholas School of the Environment, Duke University Marine Laboratory, 135 Duke Marine Lab Rd, Beaufort, NC 28516 USA

**Keywords:** Geography, Environmental sciences

## Abstract

Shipwrecks hold significant historical, archaeological, and ecological value. In this dataset, we present two high-resolution (~0.60 cm & 3.0 cm GSD) orthomosaics and associated data that accurately maps the so-called “Ghost Fleet of Mallows Bay”, a prominent shipwreck assemblage near the eastern banks of the Potomac River, Maryland, USA. Using unoccupied aircraft systems (UAS), we conducted aerial surveys at regional and individual wreck scales, imaging all 147 wrecks in the bay. Through structure-from-motion photogrammetric processing, we generated the highest-resolution georeferenced mosaics currently available for Mallows Bay. We used the regional orthomosaic to vectorize individual wrecks, with the resulting polygons linked to archaeological records from the Maryland Historic Trust. These data establish a baseline for the shipwreck-associated ecological, archaeological, and cultural resources at Mallows Bay. The orthomosaics and associated outputs suit various applications, including image analysis and habitat mapping. The digital spatial records of individual wrecks support field research efforts and aid in monitoring the evolution of shipwrecks over time.

## Background & Summary

**Fig. 1:**
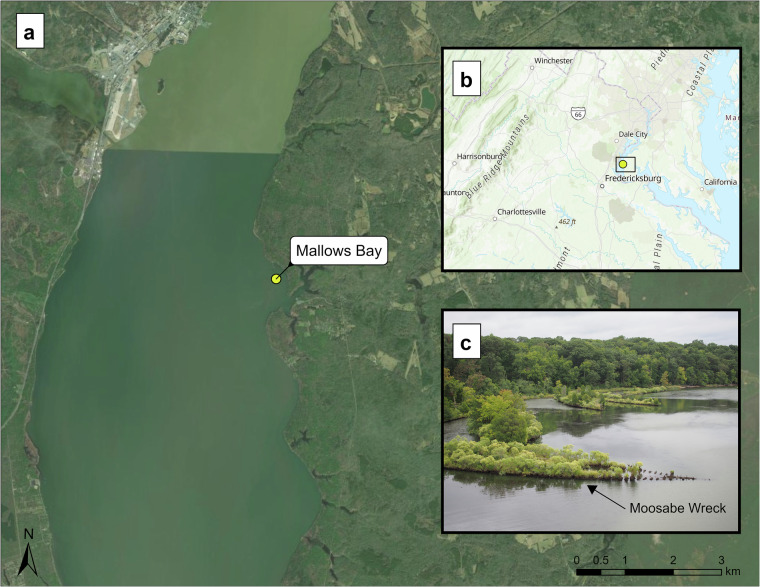
Location of the “Ghost Fleet of Mallows Bay”, Maryland: (**a**) Location of Mallows Bay in the Potomac River (**b**) Location of Mallows Bay at a larger scale (**c**) Image obtained during the survey overlooking multiple wrecks in the bay (Marine Robotics and Remote Sensing Lab). Coordinate System: WGS 1984 UTM Zone 18. Map Layer Credits: Fairfax County, VA, VGIN, Esri, TomTom, Garmin, FAO, NOAA, USGS, EPA, NPS, USFWS, Earthstar Geographics.

Along the Potomac River in Charles County, Maryland, lies Mallows Bay (Fig. 1). At 125 acres, this site is home to over 100 abandoned World War I (WWI) steamships collectively known as the “Ghost Fleet of Mallows Bay”^1^. Built between 1917 and 1919, these ships intended to enhance the United States’ ability to transport supplies and troops to European allies^2^. After the war, Western Marine and Salvage Company purchased about 230 of these ships and subsequently moored them at Widewater Bay on the VA side of the Potomac to await transfer to Alexandria, VA for salvage^1,2^. The dismantling process started in 1922 but immediately experienced setbacks including storm activity and fires^3^. As a result of navigational hazards and community concerns, a fleet of 169 ships was moved to the shallow waters of Mallows Bay by August 1929. There, the wooden superstructures of the ships were burned away to expose salvageable materials^3^. Charles County locals continued salvage efforts during the Great Depression but as scrap metal prices increased when World War II began, a final large-scale salvage effort took place by Bethlehem Steel^1,3,4^. However, Bethlehem Steel terminated the project in 1944 and twenty years later, Idamount Inc., a development firm, alongside the Army Corps of Engineers started investigating the wrecks for a proposed real estate development project^3^. However, the removal was halted as a result of environmental concerns and a violation of state disclosure laws^1^. Over the last century, some ships floated away, while others were buried beneath sediment. Now known as the largest shipwreck assemblage in the Western Hemisphere, over 100 of the 169 WWI ships brought to Mallows Bay remain^3,5^.

Since their arrival, the ships have become an integral part of the ecology at Mallows Bay. Not only do shipwrecks hold important cultural and historical resources, but they also present ecological resources as they become important habitats for a variety of species^6^. In the Potomac, large beds of submerged aquatic vegetation in this area provide important feeding and nursery habitat for a variety of birds, fish, reptiles, amphibians, invertebrates, and mammals^4^. By decreasing erosion rates and increasing accretion rates, the ships have created plentiful wetlands, forests, and aquatic habitats^4^. Sediment collected within wrecks has created ship-shaped islands, allowing both aquatic and terrestrial vegetation to flourish (Fig. 1c). Birds, such as Osprey (*Pandion haliaetus*), nest within this vegetation and on exposed areas of ships, while aquatic organisms such as the endangered Atlantic sturgeon (*Acipenser oxyrinchus oxyrinchus*) use the subaqueous wreckage as foraging and nursery grounds^4,7^.

In addition to its ecological importance, Mallows Bay also holds significant cultural and historical resources. Indigenous sites dating back 12,000 years have been identified, and the bay is an area of significance for the Piscataway Conoy Tribe^8^. Mallows Bay is also part of the traditional lands of the Piscataway Conoy Confederacy and Sub-Tribes, the Piscataway Indian Nation of Maryland, and the Patawomeck Indian Tribe of Virginia^4,8^. Because of the important cultural, historical, and ecological resources Mallows Bay holds, the National Oceanic and Atmospheric Administration (NOAA) designated the area as a National Marine Sanctuary in 2019^4,8^. However, sea level rise, sediment infill, plant colonization, and physical deterioration are changing the nature of these shipwrecks over time^9^. Because of this, high-resolution imagery is essential for documenting their location and configuration, creating a baseline for any future cultural, archaeological, geological, and ecological studies. Unoccupied aircraft systems (UAS), or drones, are an ideal tool for this as they provide fast, safe, effective, high-resolution data that can be recreated over time to effectively monitor any changes in the environment.

Data collected from UAS are invaluable for digital historical preservation. UAS’s ability to access hard-to-reach areas ensures comprehensive preservation of cultural heritage, even in fragile or remote locations. Historical information and documentation such as photographs, videos, and drawings may not be as detailed as necessary whereas UAS allows for extremely detailed historical preservation of large landscapes, such as Mallows Bay^10^. Therefore, there is immense value in using UAS to contribute to archaeological and historical records, in addition to data from satellites and in-field assessments^11^. The use of UAS allows for the creation of detailed orthomosaics and digital surface models, which provide valuable baseline data for archaeological, geological, and ecological assessments. UAS surveys and products can contribute to digital historical preservation, allowing records to be kept well into the future.

Through this collaborative data collection effort, between historians, ecologists, and archaeologists, we documented the condition of the “Ghost Fleet of Mallows Bay” using UAS and connected these geospatial data with historical records of individual ships. In this paper, we present the original raw data and high-resolution photogrammetric products of Mallows Bay. These data and products will enable researchers to monitor and study the changing terrestrial and aquatic ecosystems of the “Ghost Fleet of Mallows Bay”, as well as the condition and movement of ships.

## Methods

In 2016, Duke University’s Marine Robotics and Remote Sensing lab employed three different UAS, each optimized for a specific task. A fixed-wing UAS collected regional-scale imagery of the entire Ghost Fleet, while a medium-sized multi-rotor UAS focused on mapping an individual wreck. Lastly, a small quad-rotor UAS collected fine-detail video footage of certain wrecks. We processed the imagery through structure-from-motion software to create orthomosaics, digital surface models, and digital elevation models at a regional and single-ship scale. To create an up-to-date map of shipwreck locations, we used the regional orthomosaic in conjunction with archaeological records.

### Data collection with UAS

Our team conducted two distinct aerial surveys over Mallows Bay, MD on September 20th, 2016 in addition to video cataloguing specific wrecks. Before executing flights, during the planning process, the flight team coordinated directly with the Federal Aviation Administration (FAA) and the United States Marine Corps at Quantico for necessary authorizations.

#### Regional mapping mission

One survey was regional and designed to image the entire Ghost Fleet, capturing all wrecks tidally exposed. We conducted the regional survey at 20:25:55 UTC (4:25 PM EST) on September 20th, 2016, corresponding to a tidal height of approximately 0.012 m MLLW (Mean Lower Low Water) (NOAA/NOS/CO-OPS tide station 8634689, https://tidesandcurrents.noaa.gov/tide_predictions.html). For this survey, we used the senseFly eBee, a 0.73 kg fixed-wing UAS propelled by a brushless electric motor and powered by a 2200 mAh lithium polymer battery. This aircraft autonomously executed pre-planned missions and triggered the camera shutter at pre-determined points. We planned and managed flights in the field using the eMotion 2 software program (https://ageagle.com/drone-software/emotion/, v 2.4.x) running on a standard laptop. The flight manager can communicate with the eBee via a radio telemetry link, allowing the pilot to actively change the flight plan or enact emergency procedures.

For the regional mission, we collected imagery using a belly-mounted Canon Powershot S110 12-megapixel digital optical camera aboard the eBee. The camera focal length was held at 5 mm, the aperture was set to f/2, shutter speed was allowed to fluctuate between 1/1000 and 1/2000 sec, and ISO was allowed to fluctuate between 100 and 1600 to compensate for shifting light levels. The eBee was equipped with orientation sensors and an onboard GPS marketed as having 2.5 m and 3.0 m horizontal and vertical accuracy, respectively. This system wrote EXIF geotags to each image collected, including x, y, and z coordinates (WGS84), heading (compass degrees), and orientation (Omega, Phi, Kappa), forming the basis for absolute georeferencing of outputs.

**Fig. 2:**
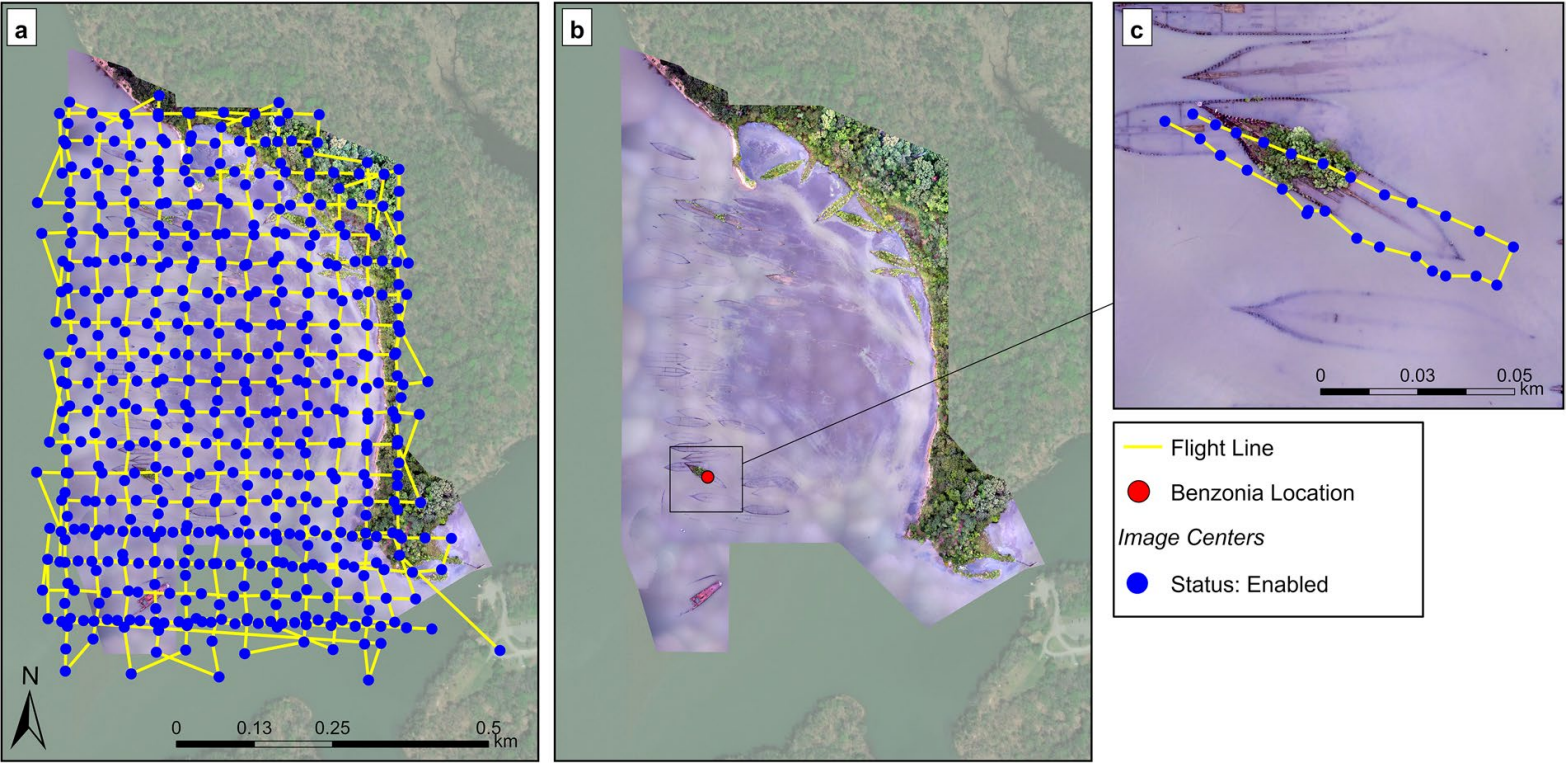
Flight track and image center locations for each survey: (**a**) Flight track over the regional area of Mallows Bay using the senseFly eBee (**b**) Location of Benzonia wreck within Mallows Bay (**c**) Flight track over the Benzonia wreck using the FreeFly Cinestar 6. Coordinate System: WGS 1984 UTM Zone 18 N. Map Layer Credits: Maxar & Microsoft.

For the regional mapping mission, we used a perpendicular grid transect design with 70% longitudinal image overlap and 70% lateral image overlap, producing 521 still images (Fig. 2a). The eBee reduces prop revolutions per minute (RPM) and adopts a slight downward pitch when triggering the camera shutter, resulting in ~7° off-nadir image orientation. The planned mission altitude was 100 m, corresponding to an average ground sampling distance of 3.51 cm/pixel. We completed the survey in two flights and the total flight time for the mission was 00:50:08 (Table 1). Table 1 provides a summary of flight parameters and conditions for both surveys.

**Table 1:** Overview of UAS surveys. This includes the aircraft, sensors, flight parameters, and flight conditions for both aerial surveys (Benzonia and Regional).

Mission	Benzonia	Regional
UAS	FreeFly Cinestar 6	senseFly eBee
Sensor	Olympus E-PM2	Canon Powershot S110
Date	9/20/2016	9/20/2016
Time (UTC)	14:52:40	20:25:55
Flight conditions	Overcast	Overcast
Total flight time (hh:mm:ss)	0:11:49	0:50:08
# of images	92	521
Maximum altitude (m)	40	100
Area covered (ha)	0.17	37.78
Average ground sampling distance (cm/pixel)	0.61	3.51

#### High-resolution imagery mission over benzonia wreck

The other aerial survey was designed at a smaller scale over the Benzonia wreck. We conducted the smaller-scale, high-resolution imagery mission with a FreeFly Cinestar 6 on September 20th, 2016 at 14:52:40 UTC (10:52 AM EST), corresponding to a 0.47 m MLLW (Mean Lower Low Water) (NOAA/NOS/CO-OPS tide station 8634689). This UAS is a 2.65 kg hexacopter propelled by six brushless electric motors and powered by two 6400 mAh batteries. Because this model was not compatible with flight planning software for mapping missions, we relied on manual pilot inputs for control via a dual stick controller and a radio telemetry link. The Cinestar 6 trades aerial endurance to fly closer to areas of interest, lift heavier sensors, and capture high-resolution imagery.

We collected imagery through a gimbal-mounted Olympus E-PM2 16-megapixel digital optical camera with a 25 mm fixed focal length lens aboard the Cinestar 6. The camera aperture was allowed to fluctuate between f/2.2 and f/2.5, shutter speed was set to 1/1250 sec, and ISO was set at 200. The camera was set to time-lapse mode, taking pictures approximately every two seconds. The Cinestar 6 was equipped with orientation sensors and an onboard GPS, which produced a log of the aircraft’s x, y, and z coordinates (WGS84) each time the camera shutter was triggered. Since the Cinestar 6 was used for fine-scale imagery and the eBee senseFly was primarily employed for mapping, the Cinestar 6 images had coarse geotagging.

Additionally, for this mission, we used a single-grid transect design (Fig. 2b,c). Because the pilot manually controlled the flight path, overlap parameters and flight lines were approximate: longitudinal image overlap was 50–70% and lateral image overlap was 75–90%, producing 92 still images during the flight. All images were in the nadir orientation. The planned mission altitude was 36 m, corresponding to an average ground sampling distance of 0.61 cm/pixel. We completed the mission in one flight, and the total flight time for the mission was 00:11:49 (Table 1).

#### Video cataloguing the ghost fleet

We also collected high resolution video from an oblique and vertical perspective of numerous wrecks including Adway, Alpaco, Arado, Bayou Teche, Buckhorn, Moosabe, The Concrete Wreck, and an unidentified wreck (MD_Site_18CH571). Using the 3DR Solo, a small quad-rotor, we obtained fine detail videos through manual flight of these prominent wrecks. Video was collected from between an altitude of 20 and 50 meters.

### Processing

#### Photogrammetric products

We processed imagery with Pix4D Mapper (https://www.pix4d.com/product/pix4dmapper-photogrammetry-software/, v 2.2.25; v 3.2.10), a photogrammetry software which uses structure-from-motion to build digital surface models and RGB orthomosaics. During the initial processing step, the software finds keypoints, or distinctive features that are detected in the images. The software then matches these points across multiple images to triangulate the camera positions^12^. Pix4D Mapper then optimizes the camera model, addressing parameters such as focal length and lens distortion to ensure accuracy. When geolocation data, such as GPS coordinates from the drone, is available, it is used to anchor the model to real-world coordinates and ensure the spatial accuracy of the outputs^12^. Automatic tie points, shared points across all the images, are created to further refine the alignment. Based on this optimized model, a dense point cloud is generated, followed by a digital surface model (DSM), orthomosaic, and 3D textured mesh. Ground control points (GCPs) were not used in processing. The output horizontal coordinate system for all photogrammetric products is WGS 1984 UTM Zone 18 N and the vertical coordinate system is WGS 1984.

Processing options differed slightly between the regional mapping mission and the Benzonia mission (Table 2). For example, alternative calibration was used for the regional mapping mission as this is optimized for aerial nadir images that have accurate geolocation^12^. We chose processing options to create the most accurate and highest resolution products. These processing details can be found in the quality reports, generated by Pix4D, which are included in the repository at the top level and labelled by the respective mission^13^. The fine-scale orthomosaic and digital surface model of Benzonia was georectified to match that of the regional scale, which had known positional accuracy, using ArcGIS Pro’s (https://pro.arcgis.com/en/pro-app/latest/help/data/imagery/georeferencing-tools.htm, v 2.0) georeferencing tools^14^. This tool allowed us to manually align Benzonia products to distinctive features on the wreck using the regional orthomosaic as a guide.

**Table 2:** Initial processing and point cloud processing options selected for photogrammetric processing of the Benzonia and Regional surveys in Pix4D Mapper (v 2.2.25; v 3.2.10).

	Benzonia	Regional
**Initial processing options**		
Image coordinate system	WGS84	WGS84
Output coordinate system	WGS84 / UTM zone 18 N	WGS84 / UTM zone 18 N
Advanced: matching strategy	Use geometrically verified matching: no	Use geometrically verified matching: yes
Advanced: keypoint extraction	Targeted number of keypoints: automatic	Target number of keypoints: automatic
Advanced: calibration	Calibration method: standard	Calibration method: alternative
	Internal parameters optimization: all	Internal parameters optimization: all
	External parameters optimization: all	External parameters optimization: all
	Rematch: auto, yes	Rematch: auto, no
		Bundle adjustment: classic
**Point cloud processing options**		
Image scale	Multiscale, 1/2, (half image size, default)	Multiscale, 1/2 (half image size, default)
Point density	Optimal	Optimal
Minimum number of matches	3	3
3D textured mesh generation	Yes	Yes
3D textured mesh settings	Resolution: medium resolution (default)	Resolution: medium resolution (default)
	Color balancing: no	Color balancing: no
Advanced: 3D textured mesh settings	Sample density divider: 1	Sample density divider: 1
	Maximum number of triangles per leaf: 8	
Advanced: matching window size	7 × 7 pixels	7 × 7 pixels
Advanced: image groups	group1	group1
Advanced: use processing area	Yes	Yes
Advanced: use annotations	Yes	Yes
Advanced: limit camera depth automatically	No	No

To generate a Digital Elevation Model (DEM) for the regional mapping mission, we first classified ground points in the point cloud generated from Pix4D Mapper using the ‘Classify LAS Ground’ geoprocessing tool in ArcGIS Pro (https://pro.arcgis.com/en/pro-app/latest/tool-reference/3d-analyst/classify-las-ground.htm, v 3.4.3). Within the tool, we used a “Standard Classification” ground detection method and “Latest” detection algorithm. We then filtered the point cloud to only ground points in ArcGIS Pro, and used the ‘LAS Dataset to Raster’ tool in ArcGIS Pro to create a DEM based on the elevation values of ground points. Specifically, we had the tool assign the average elevation value within a cell size of 1 meter (https://pro.arcgis.com/en/pro-app/latest/tool-reference/conversion/las-dataset-to-raster.htm). We also constructed a Digital Surface Model (DSM) for the regional mapping mission using the same process as above, except we included all points. We opted to use ArcGIS Pro instead of Pix4D Mapper for DEM and DSM generation because of its enhanced capabilities and visualization tools. We would like to point out that the goal of data collection was to create orthomosaics of the Ghost Fleet to understand the locations of the ships using structure-from-motion. DEMs and DSMs are distorted, because the process of structure-from-motion for point cloud generation is not optimal.

#### Digitizing ship outlines

An analyst at the Marine Robotics and Remote Sensing Lab manually digitized the ships, delineating them into polygon features in ArcGIS Pro (v 2.0)^13,14^. To guide digitization, the analyst used the “Benzonia” and “MallowsBay_Regional” orthomosaics at a 1:500 map scale^13^. The attribute table of this shapefile was populated with previously published and publicly available ship-specific information produced by the Maryland Historic Trust and Donald Shomette^15–17^. Ship information included the Maryland Historical Trust site ID, the hull design type, the construction year, and the coordinates of the ships’ center. The “Mallows_Ship_Points” shapefile was derived from the centroids of the “Mallows_Hull_Outlines” shapefile, and has the same attribute information. Spatial reference information is embedded in the shapefiles’ source metadata^13^.

## Data Records

**Fig. 3:**
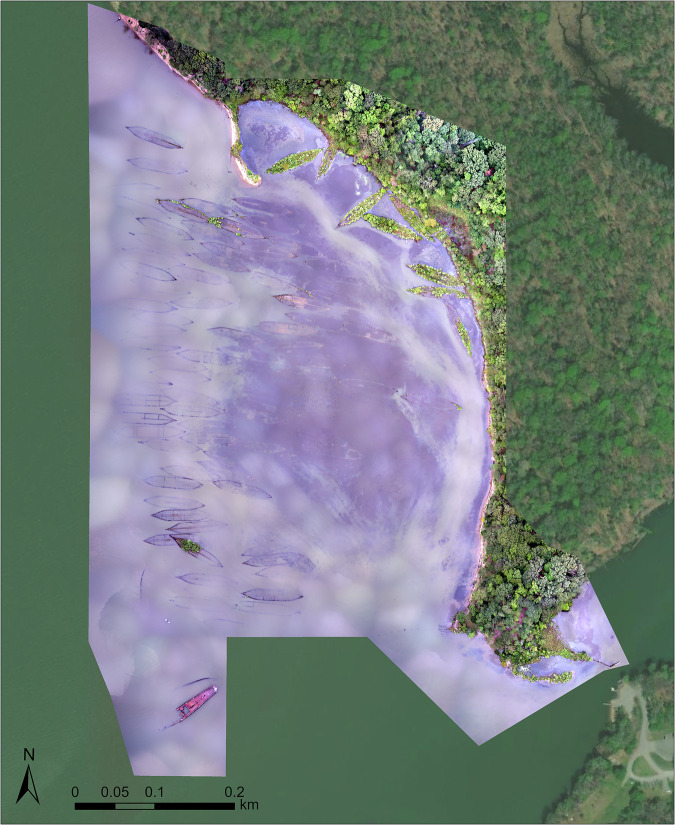
Orthomosaic of the “Ghost Fleet” generated at the regional scale of Mallows Bay. Flight conducted on September 20th, 2016 at 20:25:55 UTC with the senseFly eBee UAS. Coordinate System: WGS 1984 UTM Zone 18 N. Map Layer Credits: Maxar.

**Fig. 4:**
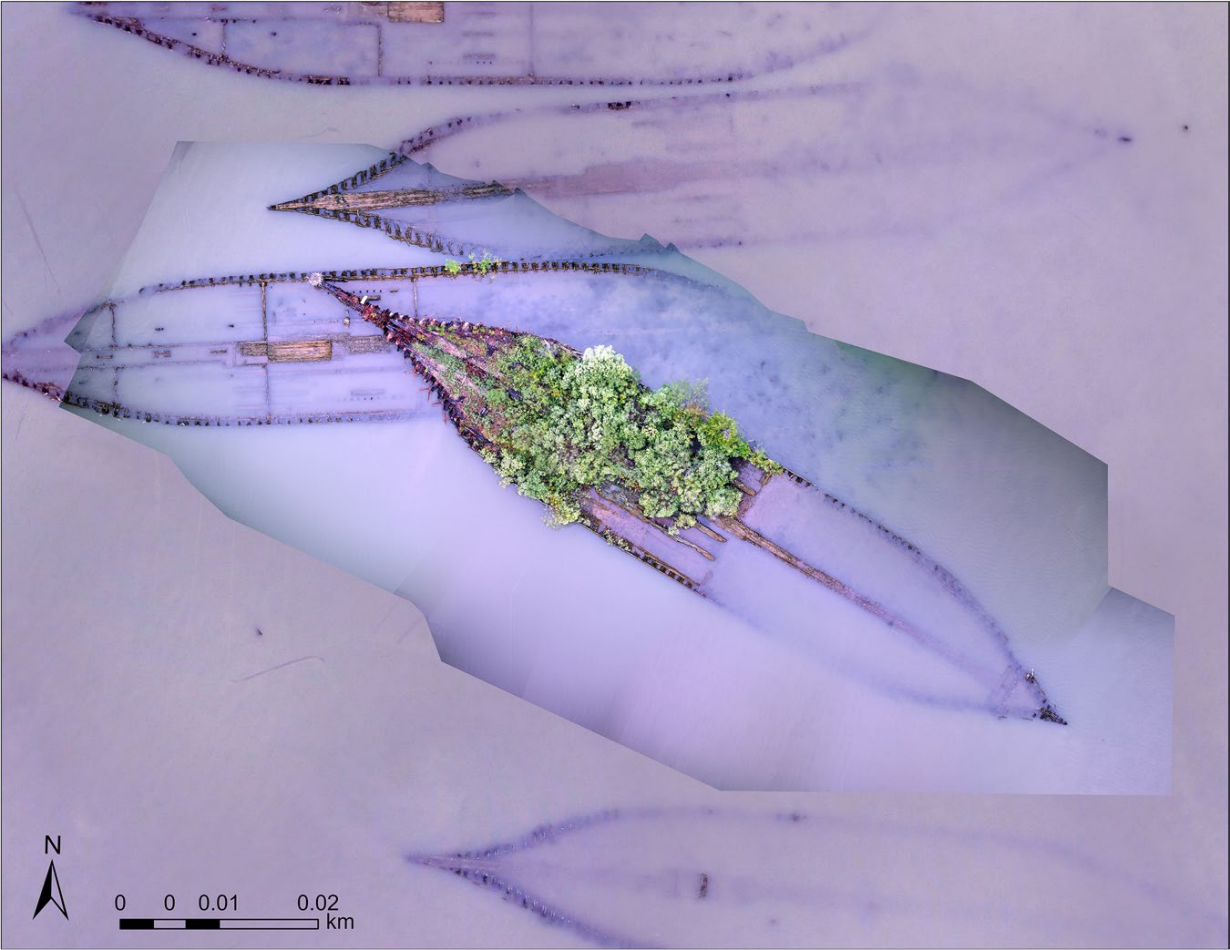
Orthomosaic of the “Benzonia” wreck generated from imagery obtained at a smaller scale. Flight conducted on September 20th, 2016 at 14:52:40 UTC with the FreeFly Cinestar 6 sUAS. Coordinate System: WGS 1984 UTM Zone 18 N.

**Fig. 5:**
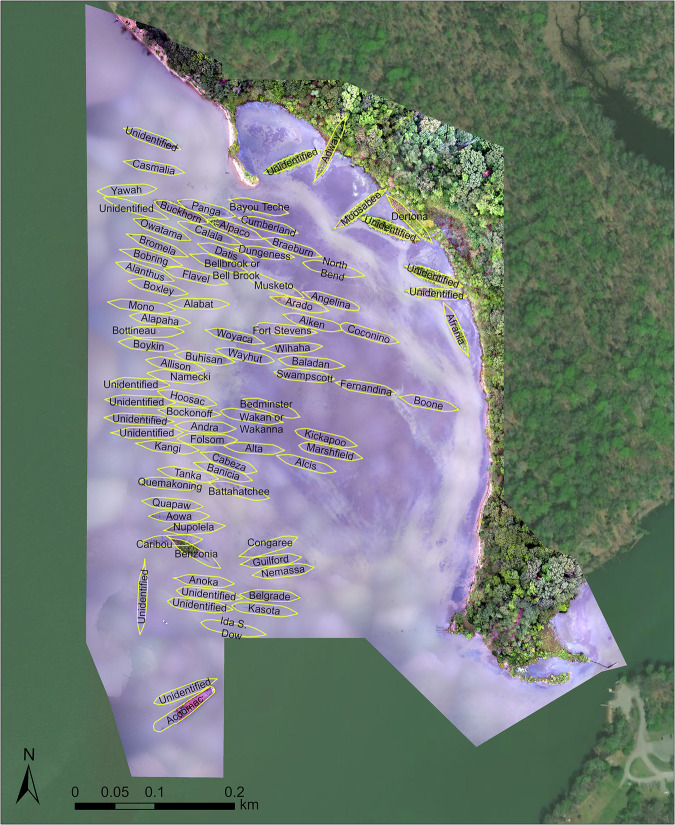
Digitized ship polygons, labelled by ship name from the Maryland Historic Trust. Ship locations are overlayed on the orthomosaic generated at the regional scale of Mallows Bay. Flight conducted on September 20th, 2016 at 20:25:55 UTC with the senseFly eBee UAS. Coordinate System: WGS 1984 UTM Zone 18 N. Map Layer Credits: Maxar.

The data is publicly available from Duke University’s Research Data Repository and includes numerous products including orthomosaics, digital elevation models and digital surface models, along with raw imagery, derived from flights over both the regional scale and an individual wreck^13^. The data is organized by mission, with data labelled by the corresponding mission (regional survey or wreck-specific flight over Benzonia). Photogrammetry products include both the regional (“MallowsBay_Regional.tif”) (Fig. 3) and ship-specific orthomosaics (“Benzonia.tif”) (Fig. 4) in a.TIF format along with the ship-specific DSM in a.TIF format (“Benzonia_DSM.tif”). A DSM and DEM for the regional mission are also included in a.TIF format (“Regional_DSM.tif”, “Regional_DEM.tif”). The ship outlines (“Mallows_Hull_Outlines”) and centroids (“Mallows_Ship_Points”) are provided in a.SHP format, which include relevant ship details in the attribute table (Fig. 5). That attribute table is also provided as a.CSV in the repository. A text document that includes coordinate information also accompanies the raw images. In addition to raw data and products, there is a detailed quality report generated by Pix4D Mapper included in a .PDF format for each mission. The quality report includes a processing summary, quality check, calibration details, bundle block adjustment details, geolocation details, initial processing details, point cloud densification details, and orthomosaic details. Note that data may be in a compressed format to ensure mandatory supplementary files are kept together.

## Technical Validation

We assessed the quality of the photogrammetric products, for both the regional and individual wreck, based on the quality report generated by Pix4D Mapper. We georeferenced the orthomosaics using the UAS’s onboard GPS, achieving an estimated absolute horizontal accuracy of 2–5 meters. Table 3 denotes the quality check derived from the quality report generated by Pix4D Mapper. The processing of the smaller scale mission over Benzonia exhibited a higher median of over 24,000 keypoints per image compared to the regional mission, indicating that the software identified more distinct features. Additionally, 86% of the images from Benzonia were successfully calibrated, compared to 68% of images from the regional mission. The matching quality was higher in Benzonia, with a median of 5,792 matches per calibrated images (Table 3).

**Table 3:** Comparison of key quality parameters between Benzonia and Regional datasets processed in Pix4D Mapper (v 2.2.25 and 3.2.10).

Mission	Benzonia	Regional
Median of keypoints per image	24,040	8,126
Images calibrated (%)	86	68
Median of matches per calibrated image	5,792.02	719.67
Georeferencing?	Yes	Yes
3D GCPs?	No	No

Between the two missions, camera optimization percentages are similar, with Benzonia at 0.2% and Regional slightly higher at 0.24% (Table 4). The mean reprojection error, which reflects the alignment accuracy between the images and the model, is lower for Benzonia (0.170 pixels) compared to Regional (0.179 pixels), indicating marginally better performance (Table 4). The reprojection error should be less than or equal to one pixel, and therefore both products had a high-quality calibration process^12^.

**Table 4:** Comparison of accuracy parameters. These include differences in camera optimization, mean reprojection error, geolocation RMS error and mean of geolocation accuracy between Benzonia and Regional processing in Pix4D Mapper (v 2.2.25 and 3.2.10).

Mission	Benzonia	Regional
Camera optimization (%)	0.2	0.24
Mean reprojection error (pixel)	0.17	0.179
Geolocation RMS error (m) (x; y; z)	2.48; 1.29; 0.31	0.93; 1.07; 1.11
Mean of geolocation accuracy (m) (images X [%]; images Y [%]; images Z [%])	5.00; 5.00; 10.00	4.00; 4.00; 4.02

However, in terms of geolocation root mean square (RMS) error, Benzonia shows larger errors in the x (2.48 m) and y (1.29 m) axes but smaller error in the z axis (0.31 m), while Regional has more balanced geolocation RMS errors across all axes (x = 0.93 m, y = 1.07 m, z = 1.11 m), suggesting more consistent spatial accuracy (Table 4). This is likely a result of the onboard GPS on the eBee senseFly used for the regional mapping mission.

Most notably, there is a marginal deviation in image calibration for the regional mission (Table 3). This is likely attributable to the structure-from-motion software’s difficulty handling low-texture areas, where object distinction is problematic, such as over bodies of water. Additionally, a slight decrease in GPS accuracy was observed for the Cinestar 6 flight over the Benzonia wreck, likely due to a lag between the camera shutter and GPS recording (Table 4). Because the regional mission had relatively better geolocation accuracy, we opted to georectify the outputs of the Benzonia mission to align with those of the regional mapping mission. We also recognize the distortions in open water regions of generated DEMs and DSMs. This stems from the fact that structure-from-motion using visible light images performs poorly over certain textures such as water, as consistent keypoint matching is limited by small waves and water movement^18^. Despite these minor discrepancies, the orthomosaics and other products are of high quality and suitable for further geospatial analysis. It is important to note that these data were collected prior to the integration of RTK/PPK (Real Time Kinematics/Post Processed Kinematics) technology into UAS platforms, which may impact overall accuracy compared to modern systems. We used legacy platforms like the eBee and 3DR, which may introduce slight accuracy constraints.

## Usage Notes

Photogrammetric products and shapefiles can be visualized using geographic information system (GIS) software, including ArcGIS Pro (Esri, https://www.esri.com/en-us/arcgis/products/arcgis-pro), QGIS (QGIS Development Team, https://www.qgis.org), R (R Core Team, https://www.r-project.org) and Python (Python Software Foundation, https://www.python.org).

## Data Availability

We processed imagery using structure-from-motion photogrammetry in Pix4D Mapper (Pix4D SA, Lausanne, Switzerland, v 3.2.10 & v 2.2.25) and ArcGIS Pro (v 2.0 & v 3.4.3) software. Specific processing details and options for re-creating photogrammetric products are located in the quality report generated by Pix4D Mapper and are included in the repository dataset in .PDF form in the relevant mission folder^13^. Processing options may differ slightly in updated versions of the software.
